# Specialized Learning in Antlions (Neuroptera: Myrmeleontidae), Pit-Digging Predators, Shortens Vulnerable Larval Stage

**DOI:** 10.1371/journal.pone.0017958

**Published:** 2011-03-29

**Authors:** Karen L. Hollis, Heather Cogswell, Kenzie Snyder, Lauren M. Guillette, Elise Nowbahari

**Affiliations:** 1 Interdisciplinary Program in Neuroscience & Behavior and Department of Psychology, Mount Holyoke College, South Hadley, Massachusetts, United States of America; 2 Laboratoire d'Éthologie Expérimentale et Comparée, EA 4443, Université Paris 13, Villetaneuse, France; Michigan State University, United States of America

## Abstract

Unique in the insect world for their extremely sedentary predatory behavior, pit-dwelling larval antlions dig pits, and then sit at the bottom and wait, sometimes for months, for prey to fall inside. This sedentary predation strategy, combined with their seemingly innate ability to detect approaching prey, make antlions unlikely candidates for learning. That is, although scientists have demonstrated that many species of insects possess the capacity to learn, each of these species, which together represent multiple families from every major insect order, utilizes this ability as a means of navigating the environment, using learned cues to guide an active search for food and hosts, or to avoid noxious events. Nonetheless, we demonstrate not only that sedentary antlions can learn, but also, more importantly, that learning provides an important fitness benefit, namely decreasing the time to pupate, a benefit not yet demonstrated in any other species. Compared to a control group in which an environmental cue was presented randomly vis-à-vis daily prey arrival, antlions given the opportunity to associate the cue with prey were able to make more efficient use of prey and pupate significantly sooner, thus shortening their long, highly vulnerable larval stage. Whereas “median survival time,” the point at which half of the animals in each group had pupated, was 46 days for antlions receiving the Learning treatment, that point never was reached in antlions receiving the Random treatment, even by the end of the experiment on Day 70. In addition, we demonstrate a novel manifestation of antlions' learned response to cues predicting prey arrival, behavior that does not match the typical “learning curve” but which is well-adapted to their sedentary predation strategy. Finally, we suggest that what has long appeared to be instinctive predatory behavior is likely to be highly modified and shaped by learning.

## Introduction

**Figure 1:**
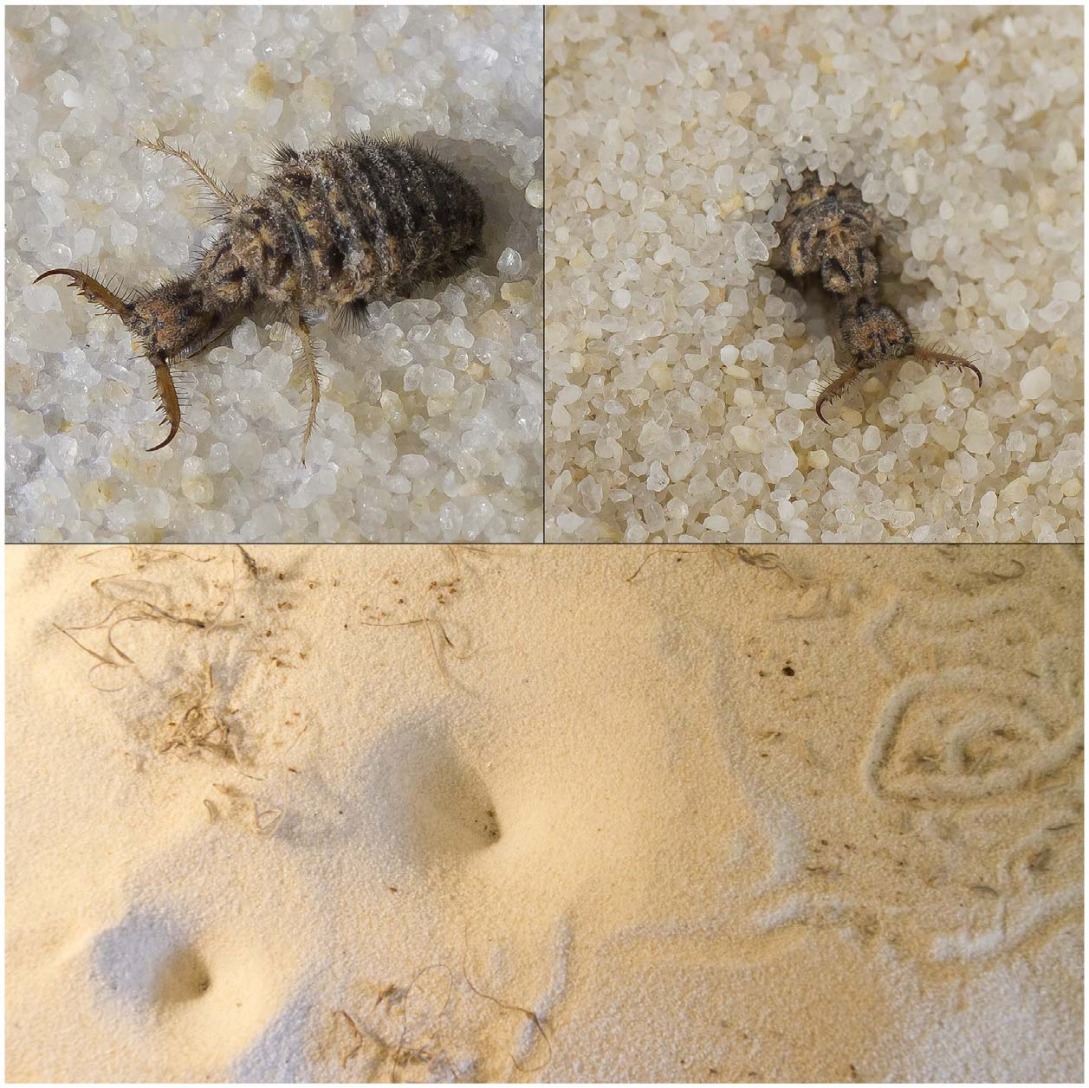
Pit-digging antlions (*Myrmeleon* sp.). A larval antlion exposed on the sand surface (top left), and in the process of burying itself under the sand (top right). Bottom: Funnel-shaped antlion pits in fine sand; the winding furrows on the right side of the photograph are the characteristic tracks made by antlions as they search for a suitable pit location. Photography by Cheryl McGraw.

Pit-digging antlions (Neuroptera: Myrmeleontidae; see Fig. 1), the larvae of winged adult insects, are thought to be the most sedentary of insect predators [1]–[4]. After larvae emerge from their eggs and find a shady location that also offers protection from wind and rain, they construct a funnel-shaped pit in sandy soil by spiraling backwards, excavating the sand with their head and mandibles [5]–[7]. Once their pits are completed, antlions position themselves at the vertex, covered either partially or entirely by the substrate, and wait motionless unless disturbed, for prey to stumble inside. Even when prey is scarce, antlions infrequently relocate their pits [8]. Indeed, relocation is constrained by so many factors, notably the high energetic costs of moving, that some species remain in the same location for months at a time, without food, until death by starvation [9]–[10]. Because of their intermittent food supply, the length of antlions' larval period is long, relative to many other insects, lasting upwards of three years [9], [11], [12]. By comparison, antlions' egg, pupal and adult stages last 30 days or less.

Antlions' extremely sedentary behavior during the long larval stage makes them unlikely candidates for learning. Although associative learning has been demonstrated in many different species of insects [13]–[16] representing multiple families in every major insect order, all the insects chosen for study throughout this voluminous literature have been those that move about their environment as they actively seek food, locate a host, evade a parasite or avoid some noxious stimulus [17]–[29]. Associative learning essentially improves the efficiency of that movement [17], [19], [21], [22], [26], [29]. Indeed, the notion that mobility of one form or another is a closely linked characteristic of learning in the wild has been recognized as an important predictor of which insect species would be expected to have evolved the capacity for associative learning [30]. However, perhaps a better reason why antlions might not be expected to rely on learning to anticipate prey arrival is that they possess a sensory system, consisting of highly sensitive mechanoreceptors located all over their bodies, that is capable of detecting, as well as localizing, potential prey approaching as far away as 6–10 cm of the pit edge [31]–[34]. This ability to localize prey at a distance enables antlions to toss sand in the direction of potential victims, a frequently observed behavior that is thought to disorient prey and increase the likelihood that it stumbles into the pit [34]. In short, then, antlions do not match the behavioral activity profile of insects already known to possess learning capabilities, and they appear to be equipped with several instinctive behavioral adaptations, not only to detect the approach of prey well in advance, but also to handle prey efficiently, thus obviating the need for learning. Indeed, in models that describe the conditions under which animals should have evolved the capacity to learn (reviewed in [35]), antlions would appear to be a prototype of those that should rely instead on fixed patterns of behavior.

Nonetheless, associative learning has been shown to provide large fitness benefits in fish [36], [37], birds [38], [39], and several insect species [19], [21], [22], [29]; thus, even if the ability to anticipate the approach of prey provided only a slight predatory advantage in antlions, relative to the costs of learning, it would have been favored by natural selection. Therefore, we explored whether the ability to associate a brief vibrational stimulus with the arrival of prey would provide a fitness benefit to pit-digging larval antlions, enabling them to pupate sooner.

We selected 19 pairs of third instar antlions, each of which was closely matched for weight, body length and pit volume. One member of each pair was randomly assigned to the Learning treatment; its pairmate was assigned to the Random (control) treatment. Antlions in both treatment groups received one prey item, delivered directly to their pits, each treatment day at the same, randomly determined time. However, for Learning antlions, a 5-sec vibratory cue – a stimulus to which antlions do not respond initially – preceded prey delivery; whereas, for Random antlions, the cue was presented at another, separate, randomly determined time. To assess the potential fitness benefits of learning, we measured differences between groups in the number of days to pupate, as well as in subjects' responses to the vibratory cue. Our results show that antlions are indeed capable of anticipating prey arrival through associative learning, which provides important fitness benefits but which does not manifest itself behaviorally with the frequency of a typical “learning curve.” Our findings not only expand our understanding of how learning benefits animals, but also force us to modify current models for the evolution of learning [35], [40], [41].

## Results

**Figure 2:**
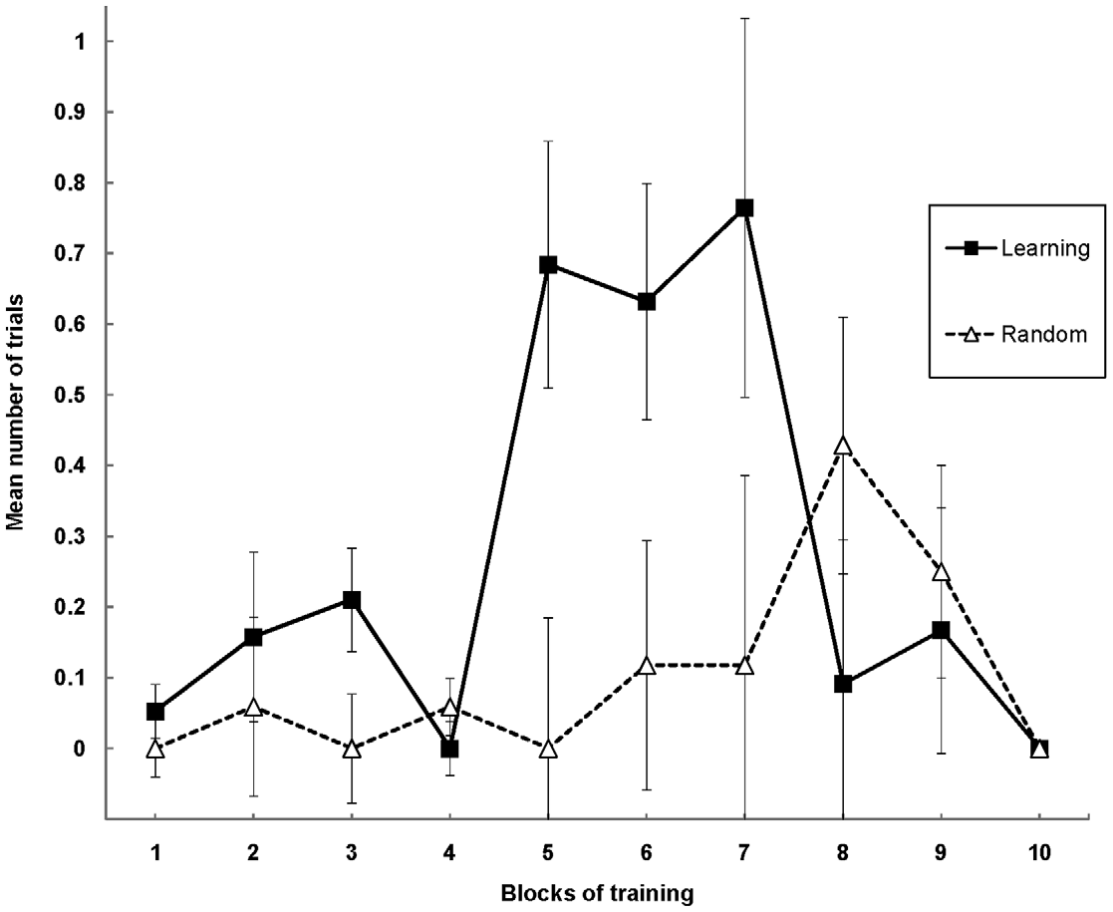
Days to pupation. Kaplan-Meier survival curves for matched pairs of Learning and Random subjects (*n* = 36). The data were analyzed using the standard statistic for Kaplan-Meier survival analysis, namely a Mantel-Cox log rank chi-square analysis. That analysis revealed that Learning antlions pupated in significantly less time than Random antlions, χ^2^ (1, *N* = 36) = 7.66, *p*<0.01. Following 70 days of treatment, 79% of Learning antlions pupated (15 of 19), while only 35% of Random antlions pupated (6 of 17). Median survival time, here median days to pupation, corresponding to the time point at which half of the animals remained (i.e., 50% cumulative survival), was 46 days for Learning antlions; median survival time was not reached in Random animals, even by the end of the experiment on Day 70.

Of the 19 pairs of subjects, two Random subjects stopped taking prey during the training period, leaving 17 Random subjects and 19 Learning subjects. Because including these Random subjects would have biased the results in favor of our experimental hypothesis, they instead were eliminated from all analyses; all remaining animals, in both groups, captured and consumed prey whenever it was made available. Kaplan-Meier survival analysis, a biostatistical technique used to examine the rate at which subjects in a study present a specific event or reach a well-defined endpoint, and which has been applied successfully in other ecological contexts [42], was used to compare the rate at which Learning and Random subjects pupated (see Fig. 2).

Survival rates of Learning and Random subjects (*n* = 36) were analyzed using a Mantel-Cox log rank chi-square analysis. That analysis revealed that Learning antlions pupated in significantly fewer days (*M* = 50.73) than did Random antlions (*M* = 62.41), a difference of 18.7% fewer days, χ^2^(1, *N* = 36) = 7.66, *p*<0.01. The often-used survival statistic, “median survival time,” which corresponds to the time point at which half of the animals remain in a given treatment, was 46 days for Learning antlions and was not reached in Random animals, even by the end of the experiment on Day 70 (see Fig. 2). Indeed, by the conclusion of the experiment, 78.9% of Learning antlions (15 of 19) already had pupated while only 35.3% of Random antlions (6 of 17) had pupated in that same time period.

**Figure 3:**
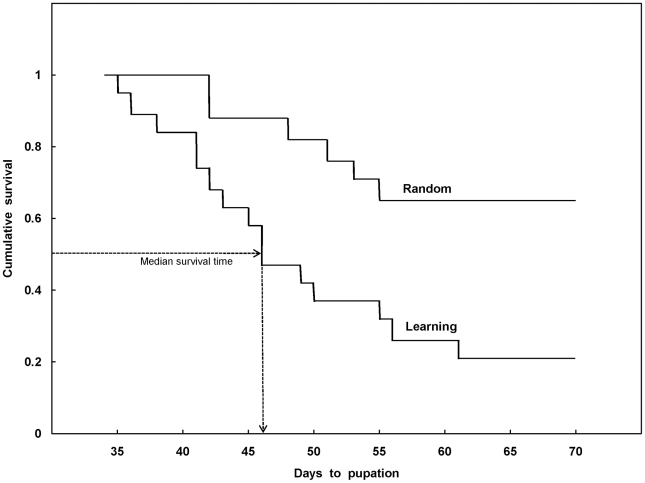
Cue-elicited sand-tossing during training. The mean number of trials (± SEM) per block in which Learning and Random subjects exhibited sand tossing behavior in response to the vibratory cue. Because each subject received 6 days of training per week until it pupated, or until the experiment concluded after 10 weeks (blocks) of training, whichever came first, a score of 6 was the maximum score a subject could obtain. However, each data point represents mean performance based only on those subjects receiving training and, thus, remaining in the experiment because they had not yet pupated.

In addition to the observed decrease in time to pupate, Learning and Random antlions also differed from one another in terms of their behavioral response to the signal. Although none of the Learning and Random antlions responded to the vibratory cue on Day 1 of training, Learning antlions responded more frequently to the cue, tossing sand in its general direction, than did Random antlions, although this behavior tended to appear primarily in the middle of the 10 blocks of training. Statistical analyses support these observations. An analysis of variance (ANOVA) with repeated measures was used to compare the number of trials in which sand tossing occurred between the two treatment groups (the between-subjects variable) across blocks of 6 training days (the within-subjects, or repeated measures, variable). That ANOVA revealed a significant difference between Learning and Random antlions in their performance of sand tossing over blocks of training (groups, *F*_1,34_ = 5.107, *p* = 0.030; blocks, *F*_9,306_ = 3.173, *p* = 0.001; Groups×Blocks interaction, *F*_9,306_ = 3.462, *p*<0.001). A closer examination, using Newman-Keuls post hoc tests, of the significant interaction between treatment condition and blocks of days, indicated that Learning and Random antlions did not differ in the frequency of sand tossing behavior at the beginning of training, namely in Blocks 1, 2 and 4 (*q*_r,360_≤1.210, NS), nor did they differ at the end of training, namely in Blocks 8 through 10 (*q*_r,360_≤3.67, NS). However, cue-elicited sand tossing occurred significantly more frequently in Learning antlions in Blocks 5, 6, and 7 of training (*q*_r,360_≥6.278, *p*<0.01), after which they responded rarely, if at all, to the cue. This drop off in responding in the last three blocks of training is not an artifact of pupation: With the exception of one Learning subject, responding stopped as many as 47 days before antlions dug under the sand to pupate (M = 13.31 days). Finally, although inspection of Figure 3 suggests that Learning antlions engaged in sand tossing more frequently than Random antlions in Block 3, a comparison of the group means using a Newman-Keuls post-hoc test, the same, very conservative, test as was used to compare all other means, suggested that Learning and Random antlions did not differ from one another, *q*_11,360_ = 2.571, *p* = 0.074. Nonetheless, regardless of how Block 3 data are interpreted, the main findings of our sand tossing analyses are unaffected; that is, Learning and Random antlions did not differ from one another early in training, they diverged toward the middle of training, and then again did not differ from one another in the last three blocks of training.

Interestingly, although Learning antlions were significantly more likely to toss sand in response to the vibrational signal than were Random antlions, its occurrence remained both sporadic and relatively rare within each individual, and thus differed from typical expressions of associative learning in other animals, insects and vertebrates alike. That is, as Figure 3 illustrates, learning in this species did not reflect the typical “learning curve” in which the observed behavior occurs with greater and greater frequency over time until it reaches asymptotic performance [43]. We return to this point below.

## Discussion

Any decrease in the time that antlions spend in the larval stage, such as that demonstrated by antlions receiving the Learning treatment, would be expected to increase fitness in two ways. One, generation time would be reduced; that is, larvae that pupated faster would become reproducing adults more quickly. Antlions' 1–3-year larval stage is by far the longest and most variable of its four life stages, with the egg, pupal and adult stages each lasting just under one month. Following pupation, adult antlions spend a mere 20–30 days as adults, during which time their primary function is to reproduce. In a very real sense, then, the length of the larval stage is the limiting factor for any potential reduction in generation time. Two, a shortened larval stage would reduce larval mortality. Pit-digging antlions are especially vulnerable during their long larval period [12], succumbing to abiotic factors (high temperatures, primarily), as well as biotic factors (i.e., predation from birds and other insects, potential cannibalism when pits become too close and antlions encounter one another, and starvation). Thus, a shortened larval stage would help to attenuate these sources of larval mortality.

Although the exact physiological mechanism that triggers pupation has not yet been identified, larval growth is understood to be a critical factor [11], [44]. Not surprisingly then, in a field study in which a very modest food supplement was provided to third instar antlions – notably the same larval stage as antlions in our experiment – time to pupation decreased significantly [11]. It is important to remember that, in the present study, antlions in the Learning and Random treatments received exactly the same amount of food, at exactly the same time, each treatment day. Moreover, all antlions, in both Learning and Random treatment groups, captured and consumed each prey item whenever it was made available. Thus, our results suggest that Learning antlions somehow were able to utilize that food more efficiently than antlions receiving the Random treatment. One possibility is that the signal for food arrival elicited the release of digestive enzymes in Learning antlions, as has been demonstrated recently in cockroaches [45]. Cue-elicited enzyme release is well-known in vertebrates, of course, as the prototypical Pavlovian, or classical, conditioned response, a response long recognized to result in increased caloric extraction [46], [47]. Another possibility is that Learning antlions somehow were more efficient handling prey. We hasten to add, however, that our prey delivery protocol, described below, insured that prey capture was nearly instantaneous in subjects of both groups. Nonetheless, Learning antlions may have engaged in slightly different behavior vis-à-vis their prey, behavior that we were unable to detect through observation, such as that occurring while antlions were extracting prey contents under the sand.

Although cue-elicited sand-tossing behavior in Learning animals, which occurred sporadically during training, did not reflect the typical “learning curve” in which the observed behavior occurs with greater and greater frequency over time until it reaches asymptotic performance, this pattern appears to be well-adapted to this extremely sedentary species: Sand tossing, like pit construction, is metabolically costly and thus should be a greatly conserved behavior. Indeed, even in those Learning antlions that responded most frequently to the cue, the typical pattern was to toss sand two or three days in a row, stop responding for several days, resume performing the learned response, and then cease sand tossing altogether. Although one might argue that this pattern merely reflects the fact that tossing sand did not affect the likelihood of prey capture in our preparation, this lack of instrumentality is characteristic of most associative learning studies in which a cue is paired with some biologically relevant event whether or not the subject responds to the cue (i.e., a typical Pavlovian, or classical, conditioning procedure) [35], [36], [37]. In those studies, and very much unlike Learning antlions in this experiment, animals in the learning group exhibit the typical learning curve. Research currently is underway to explore the moment-to-moment conditions under which this learned response does and does not appear.

The vibrational cue that we used in our experiment might at first seem artificial; however, we suggest one possible scenario that is not so very different from our laboratory conditions: In short, antlions may be able to detect substrate-borne vibrations at longer distances than those vibrations to which they react with observable motor behaviour [31]–[34]. That is, if antlions could detect the vibrations generated by prey while still far away – too far away to make sand-tossing effective and, thus, too far away to elicit what might be a hard-wired response to prey – then these distant vibrations might serve, via associative learning, as learned signals, readying antlions for a potential capture attempt as the prey moves closer, and preparing them in other ways, perhaps by releasing digestive enzymes as we mention above, or enabling them to orient with maximum efficiency to the direction of substrate vibrations caused by prey.

Finally, antlions' ability to learn about, and respond in anticipation of, prey arrival raises important questions for the evolution of learning. On the one hand, environmental predictability (or its converse, environmental stability) is posited to be a key variable in whether learning is expected to evolve in a particular species [35], [40], [41], [48], a view bolstered by careful experimentation and argument [41], [48], [49]. However, consensus also is building for a very different view of learning, namely that all animals possessing a nervous system should be able to learn [15], [50]. Indeed, as Greenspan [50] has suggested, learning may be a “fundamental principle of brain functionality (p. 649).” This view no doubt is fueled by the ever-increasing number of insect species, as well as other invertebrates, shown to be capable of associative learning, as well as by neuroscientists' greater understanding of neural architecture [16].

The addition of antlions to the list of insects capable of learning is especially noteworthy: As our research with this species demonstrates, learning can play a critical role in an animal long regarded as exquisitely adapted to a sit-and-wait lifestyle, an animal that never searches for food but, instead, relies on a highly sensitive sensory system to detect approaching prey, and fixed responses to capture it. In short, it's hard to imagine a better prototype for animals that are not expected to have evolved the capacity to learn. Thus, at the very minimum, the question no longer can be which *species* – or, even, which behavior systems within each species – reflect a model's predictions regarding environmental stability. Instead of assuming, even implicitly, that non-learning is the default condition and asking what conditions might favor its evolution, we instead might ask what conditions favor restrictions on this kind of behavioral plasticity, restrictions that effectively preempt the predisposition of all nervous systems to learn.

## Materials and Methods

Prior to the experiment, each antlion, obtained from AntLionFarms.com (Pensacola, FL) and housed in a small round plastic bowl (4×15 cm diam.) filled with fine sanitized Estes Marine Sand, was fed two wingless fruit flies daily until it stopped feeding and disappeared under the sand to molt. From those antlions that re-emerged as third instar larvae, 19 pairs of subjects, closely matched for weight, length and pit volume, were created. Each subject was moved to a rectangular plastic container (28×17×17 cm) filled with sand to a depth of 13 cm. One member of each pair was randomly assigned to the Learning treatment; its pairmate was assigned to the Random (control) treatment. Antlions in both treatment groups received one prey item per treatment day at the same, randomly determined time between 09:30 h and 16:30 h. During this training period, each prey item was approximately 1/4th of a live mealworm larva, cut from the head end, a procedure that enabled us to provide Learning and Random subjects with prey of virtually identical mass over the course of the experiment. In addition, to insure that prey capture time did not differ between Learning and Random antlions, the mealworm head was dropped directly into the pit, a few millimeters from the vertex so as not to hit the antlion. With this procedure, capture was instantaneous.

For Learning antlions, a 5-sec vibratory cue preceded prey delivery; for Random antlions, the cue was presented at another, separate, randomly determined time between 09:30 h and 20:30 h, but not within 4 hours of prey delivery. Random antlions received the cue equally often before and after prey delivery. The vibratory cue was produced by releasing 4.5 ml of sand from a plastic pipette, which was held in place just above the surface of each antlion's container and 4.5 cm from the center of the pit; the sand fell directly into a narrow cylindrical pipe, the bottom of which consisted of a thin plastic membrane, better to conduct the vibration. This cue, as well as its distance from the center of the pit, was chosen not only because the literature suggested that antlions would be able to detect this vibratory stimulus, but also, equally important, because the cue did not already elicit any predatory behavior, or movement of any kind, in preliminary analysis. Each antlion received its specific treatment, Learning or Random, 6 days each week until it disappeared under the sand in preparation for pupating. The experiment was terminated after 10 weeks (70 days). We measured differences between treatment groups in antlions' responses to the vibratory cue and in the number of days to pupate.

A Kaplan Meier survival analysis is used on quantitative data measuring the time from a well-defined time origin, here the start of the experiment, until the occurrence of some particular event of interest or end-point, here pupating. A survival analysis differs from non-parametric tests, like a chi-square Goodness-of-fit test, because it accounts for censored data, cases in which the critical event, here pupating, has not yet occurred. In the current study, censored data was solely the result of subjects that had not yet pupated to finish the experiment.
