# Correction: Specialized Learning in Antlions (Neuroptera: Myrmeleontidae), Pit-Digging Predators, Shortens Vulnerable Larval Stage

**DOI:** 10.1371/annotation/06bf5d20-9308-4502-ae52-05cb16ac5f11

**Published:** 2011-04-26

**Authors:** Karen L. Hollis, Heather Cogswell, Kenzie Snyder, Lauren M. Guillette, Elise Nowbahari

Figures 2 and 3 were switched in production. The image listed as Figure 3 is actually Figure 2, and the image listed as Figure 2 is actually Figure 3. The legends are correct. 

